# The neural underpinning of religious beliefs: Evidence from brain lesions

**DOI:** 10.3389/fnbeh.2022.977600

**Published:** 2022-10-06

**Authors:** Irene Cristofori, Shira Cohen-Zimerman, Joseph Bulbulia, Barry Gordon, Frank Krueger, Jordan Grafman

**Affiliations:** ^1^Institute of Cognitive Sciences Marc Jeannerod CNRS, UMR 5229, Bron, France; ^2^University of Lyon, Etablissement 1, Villeurbanne, France; ^3^Cognitive Neuroscience Laboratory, Brain Injury Research, Shirley Ryan AbilityLab, Chicago, IL, United States; ^4^Department of Physical Medicine and Rehabilitation, Northwestern University, Evanston, IL, United States; ^5^School of Psychology, Faculty of Science, Victoria University of Wellington, Wellington, New Zealand; ^6^Department of Linguistic and Cultural Evolution, Max Plank Institute for the Science of Human History, Jena, Germany; ^7^Cognitive Neurology/Neuropsychology Division, Department of Neurology, Johns Hopkins University School of Medicine, Baltimore, MD, United States; ^8^Department of Cognitive Science, Johns Hopkins University, Baltimore, MD, United States; ^9^School of Systems Biology, George Mason University, Fairfax, VA, United States; ^10^Department of Psychology, George Mason University, Fairfax, VA, United States; ^11^Departments of Neurology, Psychiatry, and Cognitive Neurology and Alzheimer's Disease, Feinberg School of Medicine, Chicago, IL, United States; ^12^Department of Psychology, Northwestern University, Chicago, IL, United States

**Keywords:** religious belief, cognition, social cognition, lesions mapping, dorsolateral prefrontal cortex, ventromedial prefrontal cortex

Religious beliefs emerged in conjunction with moral beliefs, political, and legal beliefs (Cristofori and Grafman, 2017). As pointed out by Oviedo and Szocik (Oviedo and Szocik, 2020) recent debates attempted to determine whether compared to other beliefs, religious beliefs have a specific (Van Leeuwen, 2014) or shared cognitive structure (Boudry and Coyne, 2016). Empirical evidence supports both positions (Levy, 2017; Van Leeuwen, 2018), while neuroscientific findings support a shared neural network between religious and other beliefs [e.g., political beliefs (Cristofori et al., 2015)]. Religious beliefs shape a person's character and influence daily decision-making and social interactions (Cristofori and Grafman, 2017). Religious beliefs are often concerned with the existence of supernatural agents and are often entangled with moral, political, economic, and legal beliefs that have collectively had a profound influence throughout known human history. Their pervasiveness and power have suggested they have a special status in the human brain. However, despite being much in need of investigation, studying the neurobiological basis of religious beliefs has proved difficult. Here, we focus on the contribution of a set of brain-lesions studies that shed light on the neurocognitive underpinnings of religious beliefs. We then compare the findings of these brain-lesions studies with results from neuroimaging studies.

## The unique contribution of brain lesion studies

Lesion studies were among the first approaches to investigating how neural-anatomy relates to brain-function (Harlow, 1848). Since the 1990 s, however, functional neuroimaging studies have come to dominate functional-anatomic brain research [e.g., (Fox and Raichle, 2007; Raichle, 2009)]. Despite their popularity, functional neuroimages studies have two limitations. First, while such studies can reveal correlations between brain activation and behavior (e.g., area X is active when behavior y is present), they are limited in their scope for providing causal understanding about the relationship (Siddiqi et al., 2021). The extent to which randomized controlled experiments may manipulate brain anatomy is clearly limited. Second, even where interventions may in principle yield causal inferential knowledge, in practice, most neuroimaging studies are underpowered [e.g., (Marek et al., 2022)]. On the other hand, the distribution of brain lesions across a population is typically a matter of chance. For this reason, by comparing religious cognition in a population with focal lesions and without lesions, causal identification if functional neuroanatomical relationships may be possible. Another virtue of functional neuroanatomical lesion studies of religious cognition, as we shall next describe, is that they are not underpowered. It is important to acknowledge that even whether brain lesions mapping is a model for studying structure/function relationship, lesions emerging from a traumatic brain injury can involve adjacent brain areas that, may accommodate different neurological and/or cognitive functions. For instance, see Rorden et al. recommendations (Rorden et al., 2009) for performing lesion behavior mapping. We do note, however, that we have studied *penetrating* traumatic brain injuries and, in that case, identification of the lesion parameters like volume loss and areas affected are made somewhat easier by the relative focality of the lesion (Raymont et al., 2011). In addition, we must highlight the studies presented here involve a slightly different cohort number, depending if the participant performed the test or not. As a reminder, our participants were tested over a 1-week period, with extensive neuropsychological and experimental assessments (for more details see Raymont et al., 2011).

## The Vietnam head injury study and its contribution for understanding the neural basis of religiosity

The Vietnam Head Injury Study [VHIS (Raymont et al., 2011)] is a longitudinal follow-up of American male combat veterans who served in the Vietnam War, most of whom suffered from focal penetrating traumatic brain injury (pTBI). This dataset provide data on participants who are similar in age, and education level, and is unique in that it includes pre-injury intelligence. The study has followed the veterans (those with, and those without, focal brain lesions) for about 50 years post injury. Particularly, in the *final phase* of the VHIS (2008–2012), 169 participants (134 with pTBI, 35 with no injury) were assessed for executive functions, social cognition, personality, as well as large battery of tests dedicated to religious beliefs, including fundamentalism, God-Image, and mystical experiences. Individuals studied here are from a monotheistic culture with one God (or no God) rather than many Gods.

## Highlighted findings

### The ventromedial prefrontal cortex and its involvement in religious beliefs

Religious beliefs can reflect the particular relationship an individual has with God. A strong personal relationship with God is theoretically (Fiori et al., 2006) and empirically (Newton and McIntosh, 2010) associated with an enhanced sense of control. A recent study by Cohen-Zimerman et al. (2020) aimed at understanding whether damage to the vmPFC—a region associated with emotionally meaningful religious experiences and with a sense of control—could modulate self-reports of a personal relationship with God and a sense of control. Voxel-based lesion-symptom mapping found that damage to the right vmPFC caused a stronger personal relationship with God, and patients with damage to this region reported a greater sense of control compared to patients with damage to the posterior cortex as well as matched healthy patients. Moreover, the association between the vmPFC damage and a greater sense of control was associated with a stronger personal relationship with God. Taken together, these results suggest that a strong personal relationship with God can serve a crucial psychological function by affecting a sense of personal control, with both enhanced after right vmPFC lesions.

More recently, Cristofori et al. (2021) investigated the neural interplay between empathy and personal relationship with God. Extending previous observations that theory of mind networks is recruited during prayer (Boyer, 2003, 2008), the authors found that people with vmPFC damage reported higher scores on the personal relationship with God inventory (Lawrence, 1997) even when they were not praying. The results showed that vmPFC and posterior superior temporal sulcus/temporoparietal junction (pSTS/TPJ) lesions, associated with the strength of the personal relationship with God, affected empathetic responses. The authors suggested that the neurological networks underpinning God representations amplify human empathetic responses. The cultural evolutionary study of religion has argued that supernatural beliefs evoke pro-social responses because people fear the wrath of Gods (Atran and Norenzayan, 2004). In accordance with other studies e.g., (Norenzayan et al., 2012), our findings imply that, in contrast to the focus of the evolutionary literature on punishment, greater attention should be addressed to investigating the mechanisms by which the religious belief system modulates empathetic responses to others. It may seem that a *stronger* relationship with God, based on the lesioned right vmPFC, is counterintuitive. However, a stronger relationship with God post-injury might be due to the crucial role of the vmPFC in scaling and evaluating social behavior (Moretti et al., 2009; Cristofori et al., 2015). Counterintuitive behavior changes provided by lesion-free contralateral homotopic areas are documented in the neuroscientific literature. However, recovery from severe and complex neural deficits may be more dependent upon extended neural networks rather than on a confined neural structure.

### The dorsolateral prefrontal cortex and its involvement in religious beliefs

Religious beliefs can be influenced by certain experiences, such as mystical experiences, i.e., subjectively believed encounters with a supernatural world. Mystical experiences diverge from religious beliefs, in the sense that someone can experience a mystical phenomenon even without prior religious beliefs. Cristofori and collaborators (Cristofori et al., 2016) investigated pTBI patients and healthy volunteers. Mystical experiences were assessed using the Mystical scale [M-Scale (Hood, 1975)]. The M-scale refers to mystical experiences that the people may have previously experienced (e.g., “I have had an experience that was both timeless and spaceless”). Voxel-based lesion-symptom mapping analysis showed that lesions to frontal and temporal brain regions were linked with greater mystical experiences. Such regions included the dlPFC and middle/superior temporal cortex (TC). Performing a confirmatory group analysis, the researchers found that the dlPFC lesion group reported experiencing increased mysticism. Notably, longitudinal analysis of pre-injury data (correlating with general intelligence and executive functions task performance) excludes explanations from individual differences. These findings support previous speculation linking executive functions to mystical experiences and reveal that executive functions (particularly those aspects of executive functions that depend upon dlPFC) causally contribute to the down-regulation of mystical experiences. This study provided evidence in favor of the executive inhibition hypothesis, for the emergence of mystical experiences. This hypothesis was based on previous studies where the authors observed decreased activity in the dlPFC during mystical exercises in practitioners of glossolalia [i.e., religious prayer group experiences in which individuals speak an incomprehensible language (Newberg et al., 2006)] or a reduction of cognitive resources invested in error monitoring during religious rituals (Schjoedt et al., 2013). Another neuroimaging study has shown that participants down-regulated regions in the dlPFC during prayers performed by charismatic speakers (Schjoedt et al., 2011).

Among the different aspects that characterize religious beliefs, a crucial one is the strength of the beliefs, i.e., the fundamentalism aspect. Previous research has identified the vmPFC as critical to representing fundamentalism (Asp et al., 2012). However, the means by which vmPFC regulates fundamentalism was still less certain. Zhong and collaborators hypothesized that the vmPFC represents diverse religious beliefs and that a vmPFC lesion would be associated with religious fundamentalism or the narrowing of religious beliefs (Zhong et al., 2017). To test this prediction, the authors assessed religious adherence with a widely-used religious fundamentalism scale (Altemeyer and Hunsberger, 1992). The results showed that participants with dlPFC lesions had fundamentalism beliefs similar to patients with vmPFC lesions, however, the effect of a dlPFC lesion on fundamentalism was associated with decreased cognitive flexibility and openness. These findings indicated that cognitive flexibility and openness are necessary for flexible and adaptive religious commitment and that such diversity of religious thought is dependent on the functionality of the dlPFC.

The increase of fundamentalism and diminished flexibility and openness might be related to hemisphere dominance laterality. There is evidence that the left hemisphere is focused on facts whereas the right hemisphere represents contextual information and, therefore more adapted to specific *situations* (McGilchrist, 2012). Moreover, in mental tasks, like meditation, left-brain dominance is effective in top-down regulation, while interhemispheric integration of facts and context takes place in anterior brain areas (Raffone et al., 2019). In addition, other psychological factors such as emotional support and self-efficacy (Zahodne et al., 2014) may influence diminished cognitive resources and induce increased fundamentalism to control new experiences and daily life.

## Conclusion

In summary, the lesion studies we describe above identify a network of neural substrates involved in the production of religious cognition, and clarify the functional relationship between religious beliefs, mystical experience, executive control, emotional regulation, rigidity of ideological commitments, and other features of social cognition. The following Table 1 summarizes the main results.

**Table 1:** Represents a summary of how damage to a brain region affects religious beliefs/experience, cognition/social cognition.

**Lesions**	**Religious belief/experience**	**Cognition/social cognition**
vmPFC	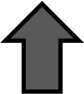 Relationship with god	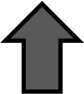 Control^*^ & empathy
dlPFC	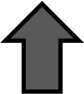 Mystical experiences	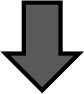 Executive functions
dlPFC	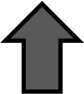 Fundamentalism	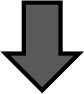 Flexibility & openness

Pointing up/down arrows indicated an increased/decreased religious or cognitive mechanism.

* The relationship with God and sense of control involved specifically the right vmPFC.

We have delineated a network of lesioned areas within the prefrontal cortex including dlPFC and vmPFC, and more posterior regions such as superior temporal cortex (STC), and pSTS/TPJ (default mode network). More importantly, the network involved included areas damaged in both hemispheres, i.e., vmPFC on the right (belonging functionally to the default mode network) and dlPFC on both sides (belonging functionally to the fronto-parietal control network). In addition, spared areas of the left hemisphere might have driven recovery, e.g., left vmPFC homotopic to its damaged right counterpart.

To sum up, the studies reviewed here complement functional neuroimaging [e.g., (Schjoedt et al., 2011)] and non-invasive brain stimulation [e.g., (Holbrook et al., 2016)] in contributing to our understanding of the religious belief system.

Our results support the belief system model proposed by Seitz and collaborators (see Seitz et al., 2018). According to the *credition* model, beliefs are the result of neural processes involving the perception and evaluation of external information, and they drive individuals' decisions. Beliefs are unique representations with imaginative and emotional content, using linguistic and memory functions by which beliefs can be expressed, stored, and recalled (Seitz, 2022). Beliefs are fundamental cognitive constructs connecting people's prior experiences with their future behaviors (Krueger and Grafman, 2017).

## Author's note

Questions concerning the Vietnam Head Injury Study can be directed to JG, jgrafman@northwestern.edu.

## Author contributions

All authors listed have made a substantial, direct, and intellectual contribution to the work and approved it for publication.

## Funding

This research was supported by the Therapeutic Cognitive Neuroscience Fund (BG). The funder played no role in the design of this study or the interpretation of its results.

## Conflict of interest

The authors declare that the research was conducted in the absence of any commercial or financial relationships that could be construed as a potential conflict of interest.

## Publisher's note

All claims expressed in this article are solely those of the authors and do not necessarily represent those of their affiliated organizations, or those of the publisher, the editors and the reviewers. Any product that may be evaluated in this article, or claim that may be made by its manufacturer, is not guaranteed or endorsed by the publisher.
